# Cobalt-Based Cathode Catalysts for Oxygen-Reduction Reaction in an Anion Exchange Membrane Fuel Cell

**DOI:** 10.3390/membranes12070699

**Published:** 2022-07-11

**Authors:** Tar-Hwa Hsieh, Yen-Zen Wang, Ko-Shan Ho

**Affiliations:** 1 Department of Chemical and Materials Engineering, National Kaohsiung University of Science and Technology, 415, Chien-Kuo Road, Kaohsiung 80782, Taiwan; thh@nkust.edu.tw; 2 Department of Chemical and Materials Engineering, National Yu-Lin University of Science & Technology, 123, Section 3, University Road, Dou-Liu City 64301, Taiwan

**Keywords:** polyimine, diethylene triamine, cathode catalyst, oxygen-reduction reaction

## Abstract

A novel cobalt-chelating polyimine (Co-PIM) containing an additional amine group is prepared from the condensation polymerization of diethylene triamine (DETA) and terephthalalehyde (PTAl) by the Schiff reaction. A Co, N-co-doped carbon material (Co-N-C), obtained from two-stage calcination in different gas atmospheres is used as the cathode catalyst of an anion exchange membrane fuel cell (AEMFC). The Co-N-C catalyst demonstrates a CoNx-type single-atom structure seen under high-resolution transmission electron microscopy (HRTEM). The Co-N-C catalysts are characterized by FTIR, XRD, and Raman spectroscopy as well. Their morphologies are also illustrated by SEM and TEM micrographs, respectively. Surface area and pore size distribution are found by BET analysis. Co-N-C catalysts exhibit a remarkable oxygen reduction reaction (ORR) at 0.8 V in the KOH(aq). From the LSV (linear-sweeping voltammetry) curves, the onset potential relative to RHE is 1.19–1.37 V, the half wave potential is 0.73–0.78 V, the Tafel slopes are 76.9–93.6 mV dec^−1^, and the average number of exchange electrons is 3.81. The limiting reduction current of CoNC-1000A-900 is almost the same as that of commercial 20 wt% Pt-deposited carbon particles (Pt/C), and the max power density (P_max_) of the single cell using CoNC-1000A-900 as the cathode catalyst reaches 361 mW cm^−2^, which is higher than Pt/C (284 mW cm^−2^).

## 1. Introduction

Fuel cell catalysts based on elements, such as Pt, require abundant surface area to contact with gas fuels. Therefore, people usually prepare nanoscale Pt by impregnating Pt onto conductive carbon black (CB) surfaces, so-called Pt/C. Consequently, reduction reactions from the Pt ions to Pt nanoparticles are usually carried out in the presence of conductive CBs, leading to Pt/C [1,2,3,4]. For convenience, certain amino organic chemicals could also behave like reducing agents to prepare Pt nanoparticles by hydrothermal methods [5,6,7], microwave-assisted heating [8,9], or often calcination [10]. However, to improve the electron transport ability (conductivity) of Pt-nanoparticles deposited on non-conductive carbonaceous substrates, calcination in the absence of air is required to convert most of the sp3-carbon substrates into the conjugated sp2-carbon system (more aromatic structures). Naturally, during the synthesis of Pt/C, amine groups containing aromatic polymers, such as polyaniline, were adopted, behaving as both chelating and reducing agents. However, thermal annealing can also generate larger Pt particles and greatly decrease the amount of surface area.

To avoid spending budgets on the preparation of conductive materials for expensive catalysts, such as Pt, non-precious metals, such as Fe, or Co-doped nitrogen-containing carbon substrates (FeNC or CoNC) [11,12], turned out to be candidates to replace expensive Pt/C. Typical works of outperformance of non-Pt metal catalysts that improve the ORR (oxygen-reduction reaction) in a cathode are described in ref. [13,14]. We have been investigating chelating iron or cobalt ions with nitrogen-containing (N-containing) compounds, which were then calcined to become metal–organic frameworks as cathode catalysts for proton exchange membranes (PEMs) or anion exchange membrane fuel cells (AEMFCs) [15,16,17,18]. In this study, we needed more robust Co ion absorbers, which can be converted into Co-doped nitrogen-containing carbonaceous composites (Co-N-C) as cathode catalysts of AEMFCs. The carbonaceous matrix acts as a conducting medium, allowing electrons generated from the anode to transport into cathode and contact with the Co active centers in the Co-N-Cs. Since the coordination numbers for Co is 6, they are not easily fixed into the carbon matrices, whose coordination numbers is 4. However, upon nitrogen doping, not only the polarity of the carbon matrix increases, but nitrogen also can chelate with Co ions and introduce them into a stable Co, N-doped carbon network after calcination.

Aromatic polyimine (APIM) with C=N bonds can firmly coordinate with Co ions, and the polymer chains can be calcined to become highly conjugated conductive carbon matrices. However, there is only one imine in a monomer unit available to chelate with Co ions. In addition, the curved nature of the polymer long molecules can hinder the approaching Co ions from contacting with the imine unit. Therefore, less Co ions are able to touch and chelate with APIM, which will significantly impair the catalytic ability of the Co-N-C catalysts. Additionally, the imine groups linked with two large aromatic rings at both ends only left limited space to accommodate the Co ions for chelation, resulting in less chelated Co ions available. 

Diethylene diamine (DETA) which owns three aminos is used to replace aromatic diamine to provide additional amine and more space to chelate more Co ions [19,20] in the preparation of polyimine, which then calcines into a Co-N-C network and acts as the cathode catalysts for AEMFC. The Co-N-C obtained is characterized by FTIR, X-ray diffraction, and Raman spectroscopy. Morphologies are studied by SEM, TEM, and HR-TEM. The electrochemical properties, including polarization (current-voltage curve) and linear sweeping voltage (LSV), are measured by rotating disk electrodes (RDE). Eventually, a membrane electrode assembly (MEA) is fabricated, and the power density of the single cell testing is recorded to evaluate the feasibility of using Co-N, doped carbon frameworks as the cathode catalysts of AEMFC.

## 2. Materials and Methods

### 2.1. Materials

Materials include DETA (Tokyo Kasei Kogyo Co., Ltd., Tokyo, Japan), TPAl (Tokyo Kasei Kogyo Co., Ltd., Tokyo, Japan), and anhydrous cobalt(II) chloride (CoCl_2_, J.T. Baker, Radnor, PA, USA).

### 2.2. Preparation of Co-N-C Catalyst

For preparation of the Co-N-C Catalyst, 1.34 g of TPAl in 100 mL alcohol, 1.70 g of PDA in 80 mL alcohol, and 1.18 g of CoCl_2_.6H_2_O in 50 mL alcohol were mixed together into one solution. The reaction mixture was stirred at room temperature (RT) for 24 h before heating up to 70 °C and was held at the temperature until almost all alcohol evaporated, followed by slow cooling to RT. The sticky product was then transferred to a Petri dish and put in an oven controlled at 80 °C for 8 h before cooling back to RT and Co-PIM was obtained.

The dry, neat PIM without CoCl_2_.6H_2_O was prepared as well for infrared (IR) characterization. Dry Co-PIM, which is used to prepare Co-N-C catalyst, was calcined to 800 °C (800, 900, and 1000 °C) at 10 °C min^−^^1^ and maintained for 30 min in an argon atmosphere before cooled back to RT. The products were dipped in 1 M H_2_SO_4_ (aq.) at 60 °C for 24 h to dissolve and remove the impurities and magnetic portion of either CoO or Co element, followed by filtration, and cleansed with deionized water and alcohol alternatively for several times before drying in a vacuum oven at 80 °C for 24 h. The acid-leached products were under second calcination at 700 °C (700, 800, and 900 °C) at 10 °C min^−^^1^, and the purging argon was replaced with a mixture of N_2_ and NH_3_ gas. The secondly calcined products were dried again in a vacuum oven at 80 °C for 24 h. The eventual sample was named as CoNC-800A-700 (or -900A-800, and -1000A-900).

### 2.3. Characterization

#### 2.3.1. Raman Spectroscopy

The Raman spectra of all samples were obtained by a Raman spectrometer (TRIAX 320, HOBRIA, Kyoto, Japan).

#### 2.3.2. Wide-Angle X-ray Diffraction (WXRD)

A copper (Cu-Kα) Rigaku X-ray source (Rigaku, Tokyo, Japan) with a wavelength of 1.5402 Å was the target for X-ray diffraction. The scanning angle (2θ), ranging from 10° to 90° with a voltage of 40 kV and a current of 30 mA, was operated at 1° min^−1^.

#### 2.3.3. Scanning Electronic Microscopy (SEM)

The morphologies of Co-N-C catalysts were obtained by SEM (field emission gun-scanning electron microscope, AURIGAFE, Zeiss, Oberkochen, Germany).

#### 2.3.4. Transmission Electron Microscopy (TEM)

Photos of the samples were taken using an HR-AEM field emission transmission electron microscope (HITACHI FE-2000, Hitachi, Tokyo, Japan); the samples were first dispersed in acetone and were subsequently placed dropwise on carbonic-coated copper grids before being subjected to electron radiation.

#### 2.3.5. Surface Area and Pore Size Measurement (BET Method)

Nitrogen adsorption–desorption isotherms (type IV) were obtained with an Autosorb IQ gas sorption analyzer (Micromeritics-ASAP2020, Norcross, GA, USA) at 25 °C. The samples were dried in vacuum at a temperature higher than 100 °C overnight. The surface area was calculated according to the BET equation when a linear BET plot with a positive C value was in the relative pressure range. Pore size distribution was determined by the quenched solid density functional theory (QSDFT) method based on a model of slit/cylinder pores. The total pore volumes were determined at P/P_0_ = 0.95.

### 2.4. Electrochemical Characterization

#### 2.4.1. Current–Potential Polarization-Linear Scan Voltammetry (LSV)

Electrocatalyst support was implemented in a three-electrode system. A round working electrode with an area of 1.5 cm^2^ was prepared as follows: Ag/AgCl, carbon graphite, and a Pt strip were used as the reference, relative, and counter electrode, respectively. The electrochemical test was performed in a potentiostat/galvanostat (Autolab-PGSTAT 30 Eco Chemie, KM Utrecht, The Netherlands) in 0.1 M KOH_(aq)_ solution, and C–V curves were obtained from 0 to 1 V at a scanning rate of 50 mV·s^−^^1^. The catalyst ink was prepared by combining 2.9 mg Co-N-C catalyst powder with a mixture of 375 μL of ethanol and 375 μL of deionized water and stirring until uniform. Subsequently, 7.14 μL of 5% D-2020 Nafion solution (Merck, Darmstadt, Germany) was introduced into the mixture as a binder, the mixture was agitated in an ultrasonic sink for 1 h, and 5μL of the obtained ink was uniformly spray-coated on the carbon paper for C-V testing.

The current–potential polarization curves obtained from the LSVs of the various Co-N-C catalysts were measured using a rotating-disk electrode (RDE: Metrohm, Tampa, FL, USA) operating at 400, 600, 800, 1200, 1600 rpm, respectively, in O_2_-saturated 0.1 M KOH_(aq)_. The reduction current densities of various Co-N-C catalysts, which were recorded at 1600 rpm with 5 mV s^−1^ scanning speed within the measured voltage range (0.0~1.2 V), were chosen for comparison.

The onset voltage (V_onset_) and half-wave voltage (V_1/2_) were directly obtained from the LSV curves (1600 rpm). The kinetic current (J_k_), which demonstrated in the first part of ORR in the LSV curve, was taken as logarithm (Log |J_K_|) vs. voltage (V) to construct the Tafel curves from which Tafel slopes were obtained. The limited reducing current density (J_red_) was defined as the current density obtained at 0 V of the LSV curves at 1600 rpm.

The diffusion current density (J_D_) was obtained from the later stage of ORR in the LSV curve at various rotating speeds (ω) which were used to calculate the Koutecký–Levich (K–L) lines according to the K–L Equation (1). The K–L lines were then used to calculate the numbers of e-transferred (n) during ORR at different voltages.

(1) $$J_{D}=0.62\times {\mathrm{AnFD}}^{2/3}\eta^{-1/6}C\surd \omega$$

A—the geometric area of the disk (cm^2^);

F—Faraday’s constant (C mol^−1^);

D—the diffusion coefficient of O_2_ in the electrolyte (cm^2^ s^−1^);

η—the kinematic viscosity of the electrolyte (cm^2^ s^−1^);

C—the concentration of O_2_ in the electrolyte (mol cm^−3^);

ω—the angular frequency of rotation (rad s^−1^);

n—the number of electrons involved in the reduction reaction.

#### 2.4.2. MEA Preparation

An X37-50RT sheet (50 μm), purchased from Dioxide Materials, Boca Raton, FL, USA, was used as the hydroxyl ion-exchange membrane. To saturate the membranes with hydroxyl (OH^−^) ions, the X37-50RT (2 × 2 cm) membrane was submerged in 1 M KOH_(aq)_ solution for 24 h. Subsequently, the treated membranes were dipped in distilled water for 15 min and were then stored in 1 M KOH_(aq)_ solution. The catalyst inks were prepared by mixing 18 mg of Co-N-C powders in 400 mg of methanol and 400 mg of deionized water, which were mechanically stirred until uniform, followed by the addition of 90 mg of 5% Sustainion^®^ XB-7 alkaline ionomer ethanol solution (Dioxide Materials, Raton, FL, USA) before stirring again until uniform. Eventually, the catalyst mixture was ultrasonicated for 1 h, followed by dropwise coating on both sides of the treated X37-50RT sheet as the anode (20% Pt/C) and cathode electrodes (2 × 2 cm), respectively, and hot pressing at 140 °C with a pressure force of 70 kgf cm^−^^2^ for 5 min to obtain the MEA.

#### 2.4.3. Single-Cell Performance Testing

The MEA was installed in a fuel cell test station to measure the potentials and power densities of the assembled single cell at different current densities using a single-cell testing device (model FCED-P50; Asia Pacific Fuel Cell Technologies, Ltd., Miaoli, Taiwan). The active cell area was 2 × 2 cm. The temperatures of the anode, cell, cathode, and humidifying gas were maintained at about 60 °C. The fuel-flowing rates of the anode input H_2_ and the cathode input O_2_ were set at 30 and 60 mL·min^−^^1^, respectively, based on stoichiometry.

## 3. Results and Discussion

### 3.1. FTIR

**Figure 1:**
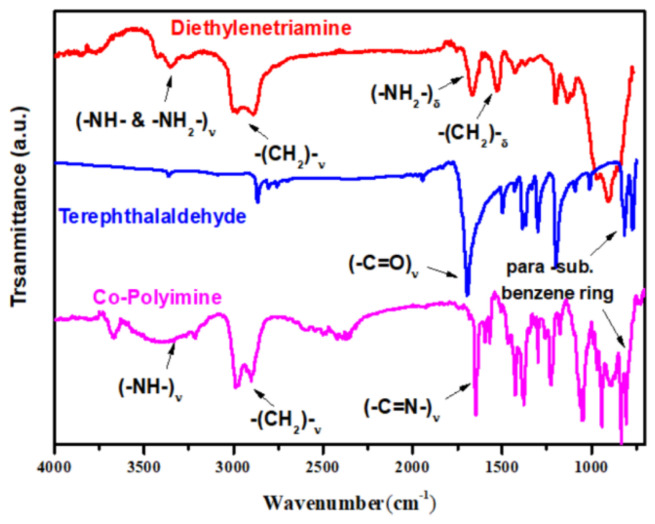
FTIR-spectra of DETA, TPAl, and co-polyimine.

Main functional group assignments of the prepared Co-PIM were based on the FTIR-spectrum demonstrated in Figure 1. The IR-spectra of each monomer (DETA and TPAl) were also obtained to compare with that of the resultant PIM in Figure 1. The stretching and bending modes of the amine and ethylene groups belonging to the DETA monomer are clearly seen in Figure 1. The amines convert to imine after condensating with TPAl, and its carbonyl group disappears as well after polymerization. The para-substituted benzene rings of TPAl and Co-PIM are found at around 819 cm^−1^. The symmetric and asymmetric stretch of the ethylene of DETA remains intact, whereas the primary amine of DETA disappears after polymerization. The wave numbers of each type of group are listed in Table 1.

**Scheme 1:**
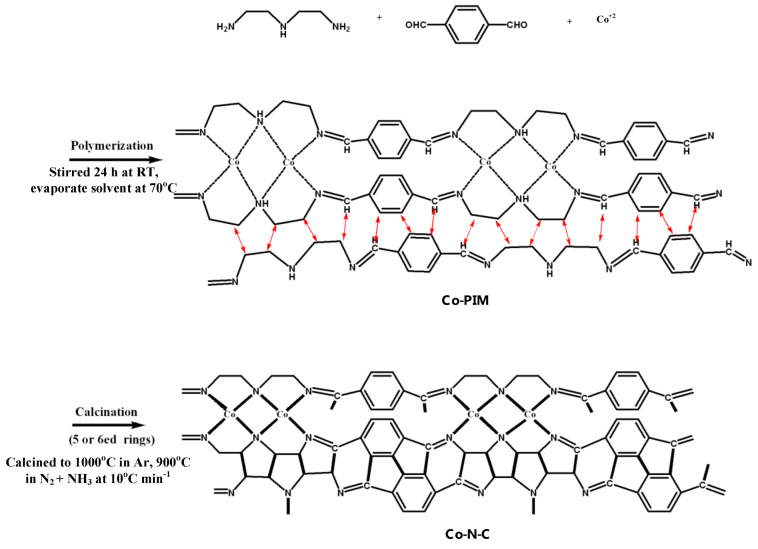
Schematic diagram of preparation of Co-PIM and Co-N-C.

An additional amine group contributed from DETA makes it more possible to cooperate with the rest of the nitrogens of imine to accommodate (complex with) more Co ions, as described in Scheme 1. The ethylene groups on the polyimine main chain introduced by the DETA are able to carry out inter-chain crosslinking since many of the five- or six-membered rings are able to form during calcination, which then develop into highly conducting Co, N co-doped (CoN_4_) carbonaceous networks of Co-N-C catalysts in accordance with the depiction in Scheme 1.

### 3.2. XRD

**Figure 2:**
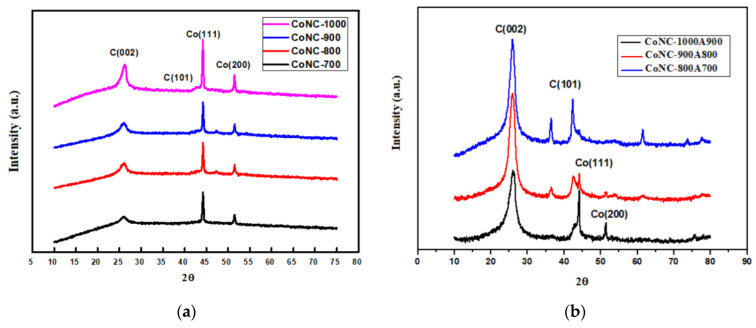
X-ray diffraction patterns of various Co-N-Cs treated with (**a**) one-stage (**b**) two-stage calcinations.

Both the Co crystalline and conjugated carbon matrix are able to develop during high-temperature calcination in the inert atmosphere, referring to Figure 2a. The intensity of (002) planes belong to either graphene (GF) or carbon nanotube (CNT) developed from conjugated carbons that grow gradually with a calcination temperature higher than 700 °C. Similarly, (111) and (200) planes of the FCC crystal of Co elements become more and more significant with temperature. It seems the Co-N-C catalysts do not experience significant oxidation in the argon atmosphere during calcination, according to the X-ray diffraction patterns demonstrated in Figure 2a. Most of the Co elements obtained in the first stage calcination in the argon, which also resulted in the magnetic attraction of Co-N-Cs that can be removed by acid-leaching, and the typical FCC planes either disappear or become insignificant compared to that of the C(002) plane in Figure 2b. However, Co crystalline was replaced with CoO gradually with increasing calcination temperatures in the second stage during which argon was replaced by N_2_ and NH_3,_ as shown in Figure 2b. Even though some CoO crystalline were developed, they are less comparable to carbon crystalline in accordance with Figure 2b or Table 2. Not surprisingly, the oxygen composition rises again (Table 2) when second calcination is carried out at 900 °C. Eventually, the carbon matrix is the dominating component, which is confirmed in Table 2 and can be confirmed by the TEM images.

### 3.3. Raman Spectroscopy

**Figure 3:**
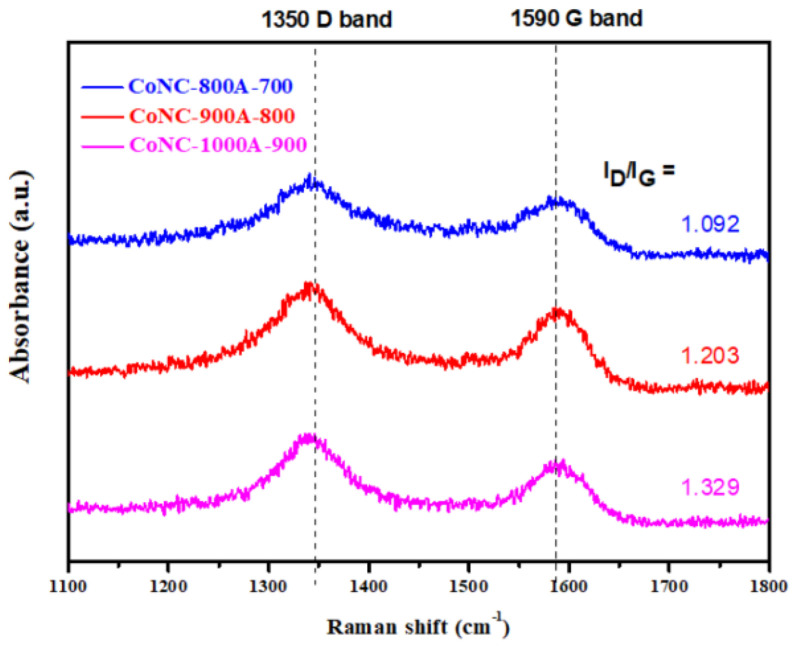
Raman spectra of various Co-N-C catalysts treated with two-stage calcinations.

During calcination, the Co-PIM carry out the carbonization process through intra- or inter-chain crosslinking and becomes a Co, N-doped network with some CoNx units present in the carbonaceous matrix, which also behave as the catalytic centers for ORR. Although Co elements and CoO developed during calcination, they can catalyze ORR as well. However, Co elements and CoO are either removed from the surface of networks by acid-leaching or embedded in the carbon matrix, losing their catalytic capability. The carbonization process also improves the formation of conjugated carbon bonding, resulting in a highly conducting carbon matrix composed of either GF or CNT. Consequently, the electrons can pass through the conducting Co-N-C matrix to perform the ORR in the CoNx centers. Conjugated bonds (Sp^2^ bonding) can be developed or degrade into non-conjugated ones (Sp^3^ bonding) during calcination, which can be monitored by the intensity ratio of the D-band (I_D_) and G-band(I_G_), obtained at 1350 and 1590 cm^−1^, respectively in Figure 3. We understand both D- and G-bands are present in the Co-N-C catalysts, and the unsaturated G-band lose to D-band when calcination temperature increases, and I_D_/I_G_ ratio increases from 1.092 to 1.329 (Figure 3). In other words, the surface of the catalyst becomes less flat and less crystallized due to the loss of planar Sp^2^ bonding with increasing temperature, indicating more surface areas are created at higher temperature calcination. The replacement of phenylene diamine with aliphatic DETA significantly decreases the numbers of Sp^2^ bonding, and all the I_D_/I_G_ ratios are higher than 1.000. BET measurements come to similar conclusions in the later discussion.

### 3.4. SEM and TEM Micrographs

**Figure 4:**
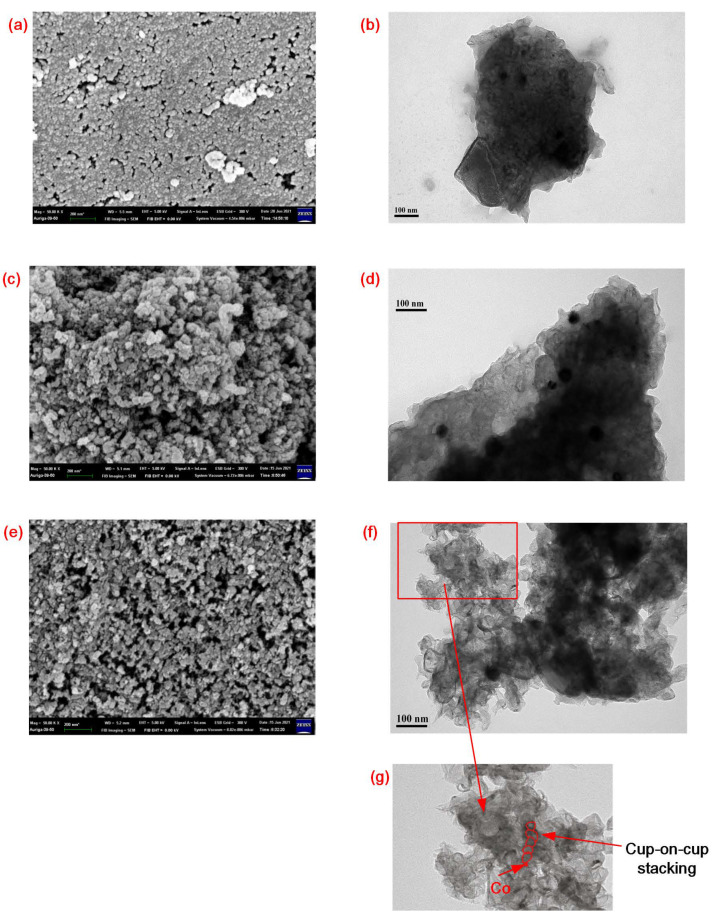
SEM and TEM micrographs of various CoNCs treated with two-stage calcinations, respectively, (**a**,**b**) of 800A-700; (**c**,**d**) of 900A-800; (**e**,**f**,**g**) of 1000A-900.

The micro-size surface morphology related to the magnitude of surface area and catalytic ability of Co-N-C catalysts can be found in the SEM micrographs in Figure 4a,c,e. We can find dense surface morphologies with fewer micro- or meso-pores for CoNC-800A-700. For CoNC-900A-800 subjected to calcination temperature with 100 °C or higher, the dense structure collapsed, and more pores formed. This indicates that bombardment of NH_3_ gas on the catalyst surface cannot generate enough pores to achieve high-surface area unless it is performed at temperatures above 800 °C (Figure 4c,e). Consequently, the surface of the CoNC-1000A-900 is filled with micro or mesopores (Figure 4e), where more CoNx centers can be exposed to the incoming O_2_ to perform ORR.

From the diffraction patterns and spectra presented in Figure 2 and Figure 3, respectively, we understand the presence of a highly conjugated carbon matrix in the Co-N-C catalyst. TEM micrographs confirmed the formation of GFs and CNTs after calcination as well in Figure 4b,d,f. When the calcination temperature is lower (CoNC-800A-700), only folded, overlapping GF sheets can be seen in Figure 4b. Some carbon materials develop into curved CNTs using Co centers as the starting seeds [21,22,23] at higher temperatures and generate more micropores and mesopores, as shown in Figure 4d,f. The inset Figure 4g clearly illustrates the formation of curved CNTs from cup-to-cup stacking of carbons [24]. The embedded Co metal or CoO inside CoNC-1000A-900 can also be found in Figure 4f or Figure 4g.

**Figure 5:**
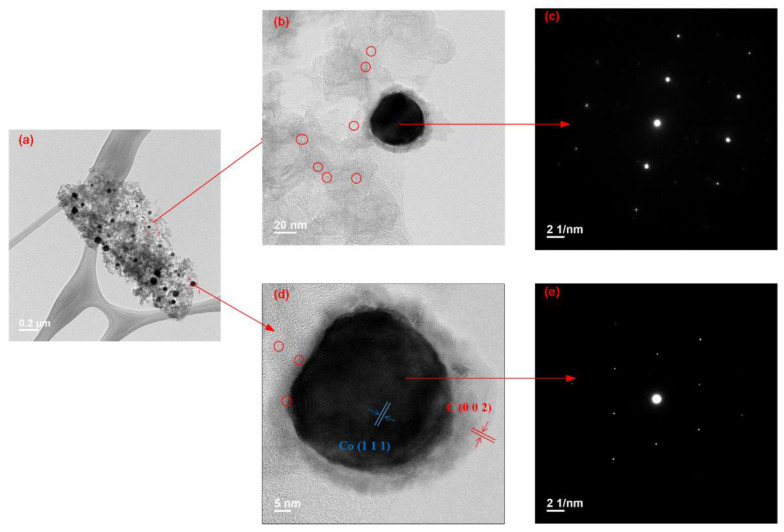
HRTEM micrographs and electron diffraction patterns of CoNC-1000A-900. (**a**,**b**,**d**): images of CoNC-1000A-900. (**c**,**e**) electron diffraction patterns of CoNC-1000A-900.

To localize the single cobalt atom [23,24,25,26,27,28,29,30,31,32,33,34,35,36] which is expressed in CoNx bonding in the carbon matrix, HRTEM pictures taken from Figure 5a at two selected sites (sites 1 and 2) are shown in Figure 5b,d, respectively. Many CoNx dots are dispersed around Co crystalline in Figure 5b and the electron diffraction pattern which is also demonstrated in Figure 5c. From the HRTEM micrograph revealed in Figure 5d, the Co(111) and C(002) planes are identified, and the electron diffraction pattern of the Co crystalline is illustrated in Figure 5e.

**Figure 6:**
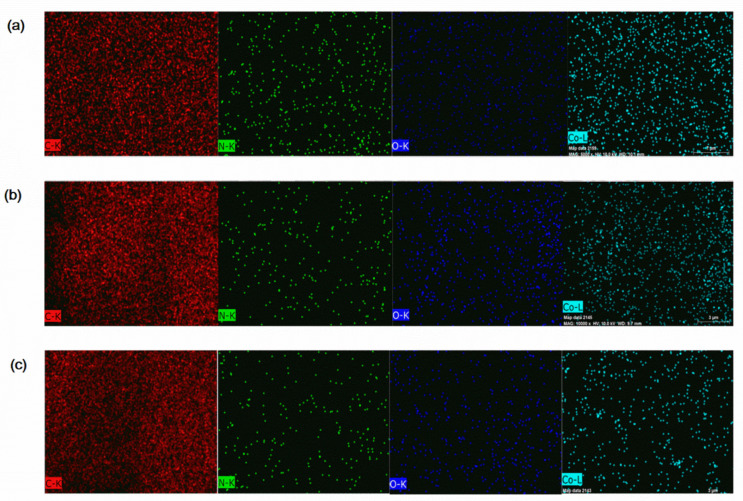
EDS mappings of Co, N, C, and O elements of (**a**) CoNC-800A-700, (**b**) CoNC-900A-800, and (**c**) CoNC-1000A-900.

The EDS mappings of the Co, N, C, and O elements were demonstrated in Figure 6 in accordance with ref [37]. The percentages of Co, obtained from the EDS spectra are listed in Table 2. Commonly, all Co-N-Cs demonstrate uniform distributions of these four types of elements, according to Figure 6. Not many Co elements are found for each Co-N-C due to the acid-leaching that removed most of Co in the form of CoO or Co metal. Only CoNx with Co covalent bonded in the catalyst network remains.

### 3.5. BET Surface Area and Pore Size Distribution

**Figure 7:**
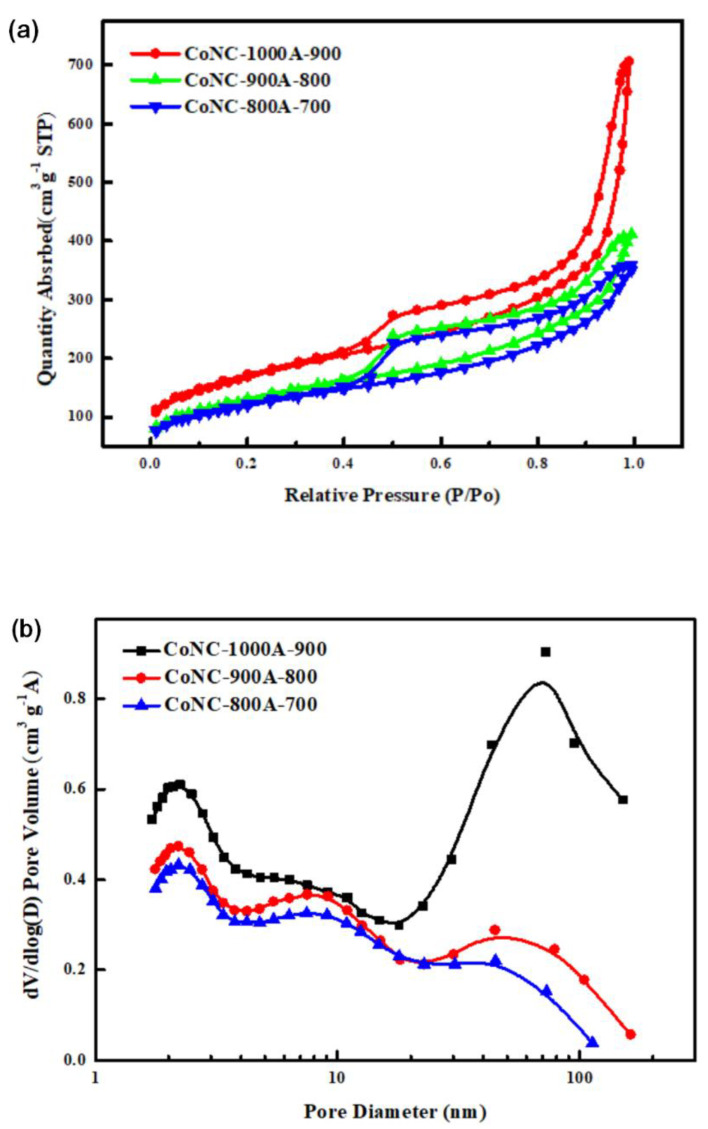
(**a**) BET isothermal adsorption and desorption curves; (**b**) pore volume vs. size of Co-N-Cs treated with different two-stage calcinations.

The morphologies seen in the SEM micrographs of Figure 5 clearly demonstrate surface area (SA) of the Co-N-C catalysts can increase with calcination temperature. We are able to obtain the quantitative SA (Table 3) from the BET isothermal adsorption and desorption curves seen in Figure 7a. All Co-N-Cs demonstrate BET SA ranged from 417 to 583 m^2^ g^−1^ (Table 3). The results listed in Table 3 also exhibit the same conclusion drawn from the observations on the SEM micrographs of Figure 5 that catalysts treated with higher temperatures can develop higher SA. The SA can even increase to be over 500 m^2^ g^−1^ (Table 3) after a second calcination at 900 °C for CoNC-1000A-900, which can contribute to the higher maximum power density of the MEA discussed in the electrochemical section. We understand the vast increase of SA for CoNC-1000A-900 comes from the formation of external (meso-) pores in accordance with Figure 7b and Table 3 due to the harsh etching capability of NH_3_ gas molecules at high temperatures. The total volume of CoNC-1000A-900 is almost twice as much as that of CoNC-800A-700 and all above 1 cm^3^, indicating more volume developing with increasing temperature. The contribution of the additional volume might result from the breaking of the aliphatic carbon single chain of DETA since the BET SA is below 400 m^2^ g^−1^, and total volume is always below 1 cm^3^ if Co, N-doped polyimine is prepared from an aromatic diamine monomer [15,16]. In other words, to replace phenylene diamine with DETA in the preparation of Co-PIM not only can chelate more Co ions by the additional amine but also provide catalysts with higher SA and volume after calcination, matching with the original purpose described in the introduction section.

### 3.6. Possible Mechanism of ORR by Co-N-C Cathode Catalyst

**Scheme 2:**
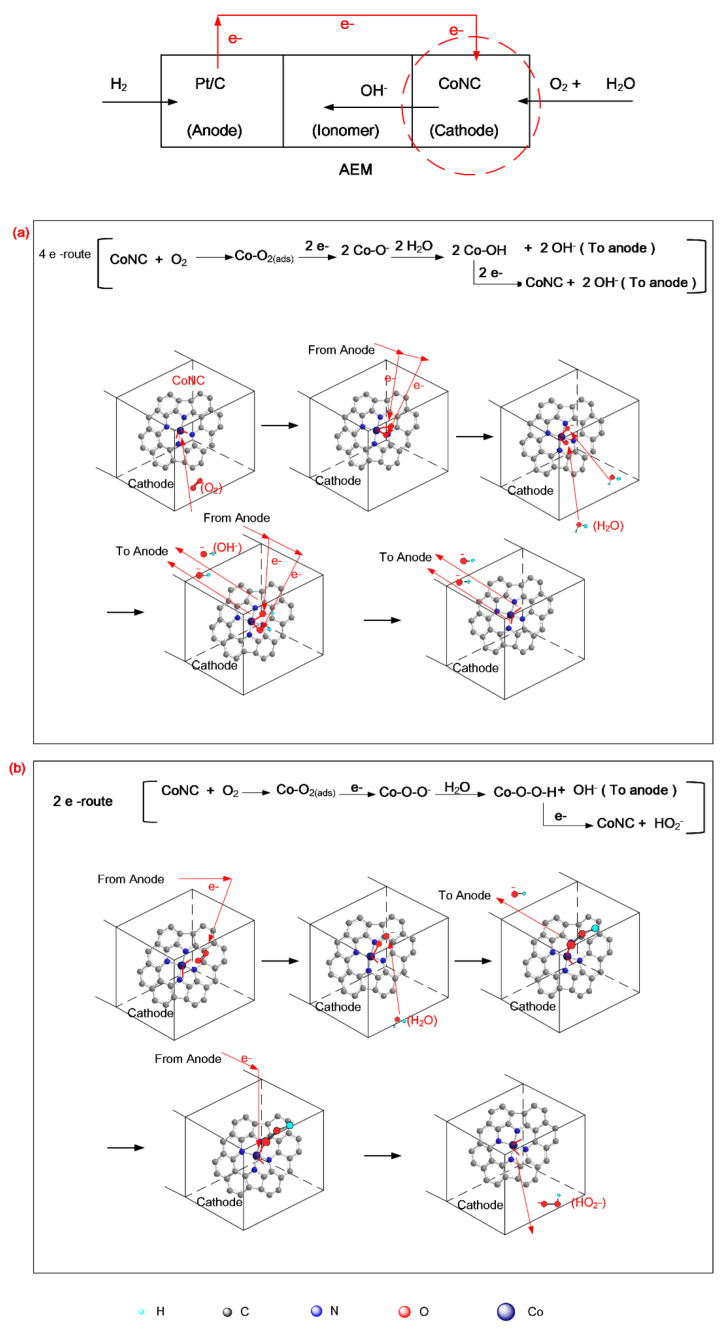
ORR mechanism catalyzed by Co-N-C cathode catalysts following (**a**) 4e^−^ and (**b**) 2e^−^route.

A schematic diagram of an AEMFC is displayed on the upper part of Scheme 2, and the ORR catalytic mechanism of Co-N-Cs in the cathode is also illustrated in Scheme 2a,b, following the 4e^−^ and 2e^−^ routes, respectively.

In Scheme 2a, O_2_ is first adsorbed by the CoN_4_ active center, the π-bond of O_2_ is broken and covalent-bonded with the empty complex sites of CoN_4_. Peroxide formed after complexation is reduced and broken into two oxygen ions (-O^−^) after obtaining the electrons donating from anode via external circuit. The strong base (negative oxygen ions) can extract the hydrogen from two water molecules and convert them to two hydroxyl ions (OH^−^), transporting to anode via the anion exchange membrane electrolyte described in Scheme 2a. These two created hydroxyl groups connected to the CoN_4_ center would break away to become two additional OH^−^ after receiving two more electrons from the anode, and the CoN_4_ center returned to its original state, preparing for the next catalytic cycle. In total, four electrons are involved in ORR, named as the 4e^−^ route described in the attached reaction equation in Scheme 2a. The net reaction (4e^−^ route) catalyzed by Co-N-Cs in the cathode is:

$$O_{2}+2H_{2}O+4e^{-}=>\;4\;{\mathrm{OH}}^{-}$$

A possible incomplete ORR can also occur when not enough electrons are coming from the anode through an external circuit or less O_2_ fuel is provided. The captured O_2_ will partially reduce to become a peroxide ion with only one electron supplied from the anode, depicted in Scheme 2b. Similarly, the strong bae (peroxide ion) can extract hydrogen from a single water molecule and becomes a hydrogen peroxide that is connected to a CoN_4_ center. The water molecule that loses one hydrogen to the peroxide ion becomes the first OH^−^ that can transport via an ionomer membrane to the anode, as described in Scheme 2b. A second electron coming from the anode can reduce the bonded hydrogen peroxide and separate into an ion (HO_2_^−^) which allows the CoN_4_ center to return to the original state in Scheme 2b. In total, only two electrons are involved and one OH^− is^ produced in this type of ORR, named as 2e^−^route. The net reaction (2e^−^ route) catalyzed by Co-N-Cs in the cathode is:

$$O_{2}+H_{2}O+2e^{-}=>\;{\mathrm{OH}}^{-}+{{\mathrm{HO}}_{2}}^{-}$$

where OH^−^ is not the only product after ORR.

Certainly, HO_2_^−^ can go on to a further reduction once an additional two electrons and one water are provided:

$${{\mathrm{HO}}_{2}}^{-}+H_{2}O+2e^{-}=>\;3{\mathrm{OH}}^{-}$$

The actual ORR in the cathode follows both 4e^−^ and 2e^−^ routes, which mean we always have the by-product, HO_2_^−^, present in the cathode, and the numbers of e- transferred (n) during ORR ranges from 2 to 4, depending on the degree of ORR into OH^−^. The n-value calculated from the electrochemical data can evaluate the efficiency of the Co-N-C catalysts.

The following discussions will focus on measuring various electrochemical properties related to the performance of the Co-N-C cathode catalysts.

### 3.7. Electrochemical Properties

#### 3.7.1. CV and LSV Measurements

**Figure 8:**
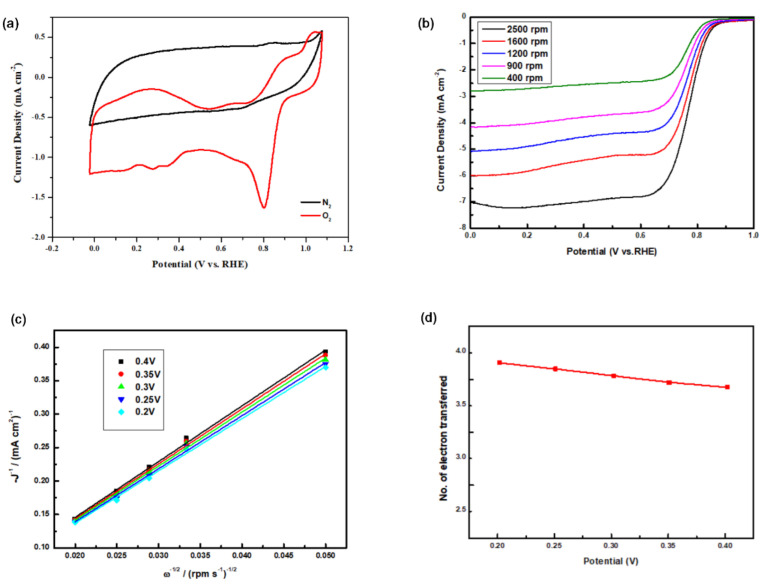
(**a**) CV-curves; (**b**) LSV curves obtained at various rpms; (**c**) K–L lines; (**d**) n-value vs. voltage of CoNC-1000A-900.

The catalysis on the ORR by CoNC-1000A-900 demonstrates a sharp reduction peak in the polarization curve (CV curve) in the O_2_ atmosphere, which is not found in the N_2_ atmosphere in accordance with Figure 8a. The CV curve measured with a static electrode at a 50 mVs^−1^ scanning rate included both reduction and oxidation processes. It was then replaced with a rotating disk electrode (RDE) at various rpms, and only the reduction current density (J_red_) was recorded with the linear scan voltammetry method (LSV) since we are studying only cathode catalysts for ORR [38]. All LSV curves of CoNC-1000A-900) illustrated in Figure 8b are measured at different rpms, producing a kinetic reduction current density (J_k_) controlled by the concentration of O_2_ in the initial stage of ORR ranging from 1.2 to 0.6 V. Eventually, the ORR was controlled by the diffusion of O_2_ gas, and the measured current is called the diffusion current density (J_D_). Based on the J_D_ obtained at various rotating speeds (Figure 8b), we are able to construct K–L lines (Figure 8c) and n-values (Figure 8d), according to the description in Section 2.4.1. The n-value measured at different voltages are all above 3.5, and the averaged n-value obtained from Figure 8d is about 3.73, very close to 4 when all ORRs followed the 4e^−^route described in Scheme 2a.

**Figure 9:**
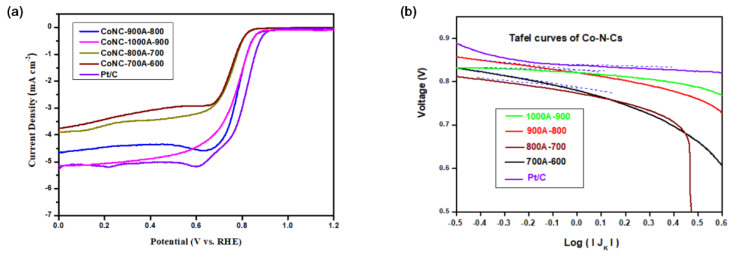
(**a**) LSV curves of Co-N-Cs treated with two-stage calcinations and Pt/C catalysts; (**b**) Tafel curves obtained from J_k_ of LSV curves.

We measured LSV curves of various Co-N-C and Pt/C catalysts at 1600 rpm and illustrated them in Figure 9a from which the onset (V_onset_), half-wave (V_1/2_) potentials, and limited reduction current (J_red_) were obtained and listed in Table 4. The Tafel reduction curves obtained from the J_k_ are demonstrated in Figure 9b from which the Tafel slopes are measured. Surprisingly, CoNC-1000A-900 demonstrate J_red_ (5.14 mAcm^−2^) comparable to that of Pt/C (5.27 mAcm^−2^) and its Tafel slope (78.4 mVdec^−1^) which is equivalent to resistance (R = V/Log(l J_k_ l)) and is the lowest as well, indicating the little resistance that was encountered during ORR. Not surprisingly, the AEM single cell that made of MEA based on CoNC-1000A-900 even demonstrates higher P_max_ (361 mWcm^−2^) than that of commercial Pt/C (284 mWcm^−2^), in accordance with Table 4, which will be discussed in the following section.

#### 3.7.2. MEA and Single Cell Testing

The discussions in paragraph of CV and LSV measurements are focused on the electrochemical behaviors of the cathode catalyst itself. However, a cathode catalyst is only part of an MEA of a fuel cell, and there are boundaries present between the electrode and electrolyte created when it is assembled into an MEA. In other words, catalysts with better electrochemical properties do not mean higher power density when assembled into a fuel cell. Certainly, we still need these measurements to find out the possible candidate catalysts for preparing MEAs. Consequently, catalyst inks were prepared and coated on both sides of the AEM (ionomers) to become an MEA for single-cell testing.

MEAs with a Pt/C anode and Co-N-Cs as the cathode catalysts were assembled for single-cell testing. An MEA was prepared with both electrodes made of Pt/C for comparison.

**Figure 10:**
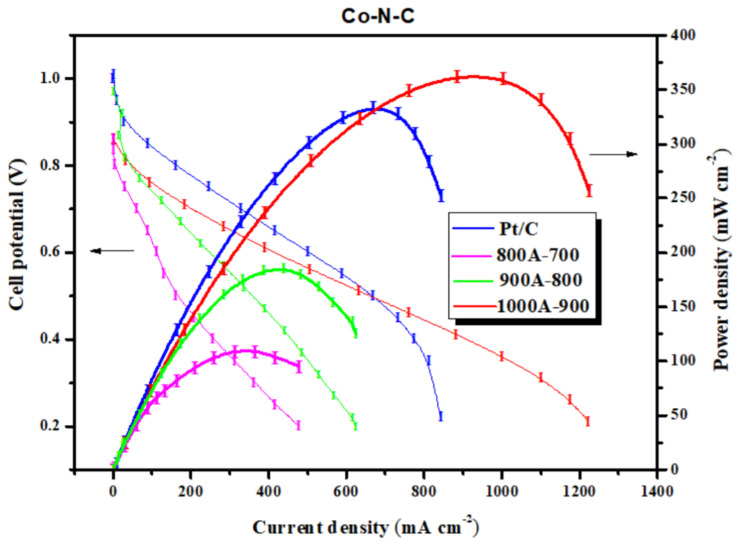
Power density and potential vs. current density of various single cells with Co-N-Cs and Pt/C as cathode catalysts.

The cell potential and power density of the single cell made of Co-N-C and Pt/C cathode catalysts are measured with the current density and demonstrated in Figure 10. Commonly, cells prepared with Co-N-C cathode catalysts do not demonstrate high open circuit voltage such as that prepared with Pt/C. However, the P_max_ of the cells prepared with CoNC-900A-800 and -800A-700 are all over 100 mW cm^−2^, which are comparable to other AEMFC with the same Pt wt% (20 wt%) in the Pt/C anode [39]. Surprisingly, the single cell made CoNC-1000A-900 cathode catalyst which was proven to own SA of 583.58 m^2^ g^−1^ and J_red_ of 5.14 mA cm^−2^, is able to contribute a higher P_max_ (361 mW cm^−2^) than that of Pt/C (333 mW cm^−2^), as shown in Figure 10 and Table 4. Additionally, its current density can be extended to be over 1200 mA cm^−2^ and that of Pt/C decay below 900 mA cm^−2^. It seems the two-stage calcination in different gases plus the acid-leach on the Co,N –co-dopes polyimine is able to build a network material with many CoN_4_ catalyst centers (most of the surface Co and CoO that are also able to catalyze ORR are already removed by strong acid-leaching). Each CoN_4_ center behaves like a single-cobalt-atom catalyst that can effectively catalyze ORR in the cathode following the 4e^−^route.

The performances of various non-precious metal catalysts are listed in Table 5. [15,16,39,40,41,42,43,44,45,46,47,48,49,50] Obviously, the P_max_ values depend on the weights of the catalysts in both electrodes and types of anode catalysts as well. This work demonstrates high P_max_ with only 20% Pt/C in the anode, compared to those using expensive Ru-Pt/C.

#### 3.7.3. Durability and Stability Test of Cathode Catalyst

**Figure 11:**
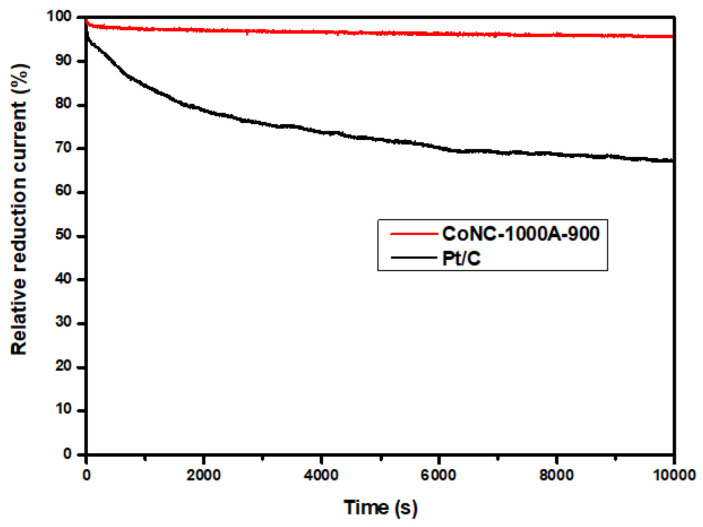
Durability test of CoNC-1000A-900 and Pt/C catalysts.

The durability and stability results demonstrated in Figure 11 were performed at the constant voltage of −0.2 V by measuring the decadence of reduction current with time at 1600 rpm. The initial reduction current was considered as 100%, and the relative current ratio was calculated by dividing with the initial current. The current decays to be 67.2% for Pt/C, while it decays only 4% for CoNC-1000A-900 (95.9%) after being treated with −0.2 V for 10,000 s as shown in Figure 11. The Co-N-C catalysts that experienced high-temperature calcination are believed to have higher durability and stability compared to Pt/C.

## 4. Conclusions

To chelate more Co ions, an additional nitrogen was introduced in the Co-N, co-doped polyimine (Co-PIM) by replacing aromatic diamine with diethylene triamine (DETA). The Co-N-C based cathode catalysts obtained from the calcination of Co-PIM exhibit remarkably high-surface area upon two-stage calcination with temperatures ranging from 700 to 1000 °C; more surface areas are created at higher temperatures. Many CoN_x_ catalytic centers were found implanted on the Co-N-C catalyst surfaces in accordance with SEM and TEM images, which mainly contribute to the ORR in the cathode. For CoNC-100A-900, it demonstrated a comparable limited reduction current to that of commercial Pt/C. Its averaged number of electrons transferred during ORR approach the theoretical value of four, following 4e^−^route ORR with negligible hydrogen peroxide ions (HO_2_^−^) formed in the cathode. The highly efficient ORR catalyzed by the Co-N-C cathode catalyst also renders the single cell prepared with CoNC-1000A-900 to generate a P_max_ of 361 mW cm^−2^, higher than that of commercial Pt/C (284 mW cm^−2^). In the future, we are planning to use multi-amino amines to prepare Co, N-co-doped carbon catalysts to illustrate the exact roles that the amines play in the preparation of efficient Co, N-co-doped cathode catalysts of AEMFC.

## Figures and Tables

**Table 1:** Assignments of main functional groups of DETA, TPAl, and Co-PIM.

Functional Groups	Assigned Wave Numbers (cm^−1^)
-NH- & -NH_2_- stretch	3424, 3363
-CH_2_-CH_2_- stretch	2987, 2903
-C=O stretch	1701
-C=N- stretch	1640
-CH2-CH2- bending	1523
para-sub. benzene ring	817

**Table 2:** Atomic ratios (%) of various Co-N-C catalysts, as determined from EDS spectra.

CoNC-Catalysts	C	N	O	Co
1000A-900	92.21%	4.59%	3.57%	0.64%
900A-800	92.36%	4.52%	2.84%	0.27%
800A-700	84.73%	8.74%	5.95%	0.84%

**Table 3:** Various surface areas (SA) and volumes (V) of Co-N-C catalysts.

Co-N-Cs	BET SA (m^2^/g)	SA_micro_ (m^2^/g)	SA_ext_ (m^2^/g)	V_micro_ (cm^3^)	V_ext_ (cm^3^)	V_tot_ (cm^3^)
1000A-900	583.58	65.76	517.82	0.24	1.92	2.18
900A-800	452.58	36.42	416.16	0.10	1.12	1.27
800A-700	417.42	41.53	375.89	0.11	1.00	1.11

**Table 4:** Comparison of various electrochemical properties of Co-N-C catalysts.

Co-N-C Catalysts	Jred ^a^ (mAcm^−2^)	V_onset_ ^a^ (V)	V_1/2_^a^ (V)	Tafel Slope ^b^ (mVdec^−1^)	P_max_^c^ (mWcm^−2^)
1000A-900	5.14	1.35	0.77	76.9	361
900A-800	4.65	1.26	0.78	78.2	184
800A-700	3.89	1.25	0.72	88.3	109
700A-600	3.75	1.19	0.73	93.6	-
Pt/C	5.27	1.43	0.80	78.9	284

^a^: obtained from LSV curves in Figure 9a. ^b^: obtained from curves in Figure 9b. ^c^: obtained from curves in Figure 10.

**Table 5:** Performance of various non-precious metal cathode catalysts of AEMFC.

Cathode Catalyst	Cathode Loading (mg/cm^2^)	Anode Catalyst	Power Density (mW/cm^2^)	Ref.
Co-N-C (PIM)	0.8	20 wt% Pt/C	361	This work
Pt/C	0.8	20 wt% Pt/C	284	This work
Co-N-C (aromatic PIM)	0.8	20 wt% Pt/C	275	[15]
Co-N-C (aromatic PIM)	0.8	20 wt% Pt/C	374	[16]
FeCoNC-at	2	Pt-Ru/C(wt% 50:25:25)	415	[39]
Co-N-CDC/CNT	0.4	Pt-Ru/C (wt% 50/25/25)	577	[40]
ZIF-CB-700	0.4	60 wt% Pt/C	122	[41]
Ag/C	0.8	40 wt% Pt/C,	207	[42]
Co–N–C	0.4	60% Pt/C	181	[43]
CoFe_2_O_4_ on Vulcan XC-72	2.4	Pt-Ru	670	[44]
N-doped CDC/ CNT	0.4	40 wt% Pt/C	310	[45]
Fe-N-Graphene	0.6	Pt-Ru/C	243	[46]
HypermecTM 4020 (FeCo-based)	0.8	40% Pt/C, 0.45 mgPt cm^−^^2^	205	[47]
CoFeN/C	0.4	46% Pt/C	177	[48]
Co-Fe_3_O_4_/C	0.8	Pt/C	114	[49]
Fe/Co-NpGr	2.5	40 wt% Pt/C, 0.80 mgPt cm^−^^2^	35	[50]

## Data Availability

The data presented in this study are available upon request from the corresponding author.
